# Biomechanical Assessments of the Upper Limb for Determining Fatigue, Strain and Effort from the Laboratory to the Industrial Working Place: A Systematic Review

**DOI:** 10.3390/bioengineering10040445

**Published:** 2023-04-05

**Authors:** Cristina Brambilla, Matteo Lavit Nicora, Fabio Storm, Gianluigi Reni, Matteo Malosio, Alessandro Scano

**Affiliations:** 1 Istituto di Sistemi e Tecnologie Industriali Intelligenti per il Manifatturiero Avanzato (STIIMA), Consiglio Nazionale delle Ricerche (CNR), Via Previati 1/E, 23900 Lecco, Italy; 2 Industrial Engineering Department, University of Bologna, 40126 Bologna, Italy; 3 Bioengineering Laboratory, Scientific Institute, IRCCS “Eugenio Medea”, 23842 Bosisio Parini, Italy; 4 Informatics Department, Autonomous Province of Bolzano, 39100 Bolzano, Italy

**Keywords:** upper limb, industrial, fatigue, strain, effort, evaluation, assessment, review

## Abstract

Recent human-centered developments in the industrial field (Industry 5.0) lead companies and stakeholders to ensure the wellbeing of their workers with assessments of upper limb performance in the workplace, with the aim of reducing work-related diseases and improving awareness of the physical status of workers, by assessing motor performance, fatigue, strain and effort. Such approaches are usually developed in laboratories and only at times they are translated to on-field applications; few studies summarized common practices for the assessments. Therefore, our aim is to review the current state-of-the-art approaches used for the assessment of fatigue, strain and effort in working scenarios and to analyze in detail the differences between studies that take place in the laboratory and in the workplace, in order to give insights on future trends and directions. A systematic review of the studies aimed at evaluating the motor performance, fatigue, strain and effort of the upper limb targeting working scenarios is presented. A total of 1375 articles were found in scientific databases and 288 were analyzed. About half of the scientific articles are focused on laboratory pilot studies investigating effort and fatigue in laboratories, while the other half are set in working places. Our results showed that assessing upper limb biomechanics is quite common in the field, but it is mostly performed with instrumental assessments in laboratory studies, while questionnaires and scales are preferred in working places. Future directions may be oriented towards multi-domain approaches able to exploit the potential of combined analyses, exploitation of instrumental approaches in workplace, targeting a wider range of people and implementing more structured trials to translate pilot studies to real practice.

## 1. Introduction

The last decade has been characterized by a revolution in the industrial sector that integrates several technologies to achieve high productivity and efficiency (Industry 5.0) [1]. The automatization of processes and the interaction between human and robot, the use of devices, and the burden of work-related diseases lead to an increasing interest in the physical and psychological state of the workers [2]. Moreover, the European Agency for Safety and Health at Work conducts the European Survey of Enterprises on New and Emerging Risks (ESENER) every four years beginning since 2009, highlighting the risks related to the workplace and, also, the psychosocial risks [3]. Industry 5.0 aims at creating a synergy between humans and autonomous machines [4], driving the transition to a human-centered and sustainable industry [1]. These recent human-centered developments in the industrial field lead companies and stakeholders to ensure the wellbeing of the industrial workers, prevent diseases and perform assessments in workplaces, assembly lines and industries, and see this as a fundamental step to improve working conditions and reduce work-related musculoskeletal disorders (WRMSD) [5]. This recent crucial step leads to an investment of resources and research mutating techniques, sensors [6] and findings from the bioengineering field, in order to apply it to the industry to enhance the industrial environments in several ways. Following this line, factors such as upper limb fatigue, strain and effort have been repeatedly measured and assessed for various purposes including, as a main target, the customization of working cells and the design of supportive devices that have effects on mental health protection, load reduction for multiple aims, including the design of supportive devices, [7] and improvements in ergonomics [8], in order to reduce work absenteeism [9] and increased well-being. These scenarios are frequently depicted in recent research projects, where several human factors are involved, including humans interaction with robots, physical and mental health monitoring in order to guarantee workers with improved working conditions and promoting workers’ good mental health, biomechanical parameters assessment [10,11], physiological measures [12], and ergonomics improvement [13]. However, since these practices have been adopted only recently, and not yet in a systematic way, we observed that often there is not a correspondence between tests made in laboratory and those carried out in working environments in real factories, leading to a gap between potential applications and those that are implemented in workplaces. Fatigue, strain and effort are generally used to refer to the physical exertion needed to perform an activity and to the associated perceived weakness and pain. Physical fatigue describes a progressive decrease in physical performance due to a prolonged sustained activity [14]; physical strain indicates an excessive physical workload during an activity that can lead to an injury [15]; physical effort refers to the use of energy needed to perform the activity [16]. Although these terms are distinct and may underlie slightly different assessments, they have a wide area of intersection and, thus, we analyzed them together in this work, referring to them with the acronym FSE (fatigue, strain and effort). Very few comprehensive reviews are available that summarize common good practice and golden standard guidelines to determine and assess workers’ FSE, and they are limited to specific scopes and very targeted fields of research. The available reviews are usually sectorial and have a more focused scope, and describe in detail specific fields, such as exoskeleton-assisted work [17], fatigue monitoring with wearable systems [18] or physical fatigue detection in construction workers [19].

Moreover, considering the large scope of industrial applications, a variety of studies, investigations and setups were implemented, considering different protocols and scenarios, as well as a variety of study designs and assessments, with very non-homogeneous approaches. Previous reviews have already summarized some of the aspects related to biomechanical fatigue in the industrial scenarios, focusing on the studies that investigated the effect of job rotation and work-rest schemes, as well as work pace, cycle time and duty cycle, on the upper limb muscle fatigue [20]. The effects of these work organization factors on subjective fatigue or discomfort were also analyzed. Electromyography (EMG) was the most used measurement, and no consistent results were found related to the effects of job rotation on muscle activity and subjective measurements of fatigue.

This paper presents a systematic review of the studies and setups specifically aimed at evaluating and assessing the motor performance of the upper limb in the industrial field. First of all, the main topics addressed by the screened studies are presented, showing their main focus on upper limb FSE. Then, a comprehensive comparison between laboratory and working settings is reported, based on the systematic screening of many relevant features including the type of enrolled participants, the type of motor task, the use of support and interaction devices and the type of assessment used. Indeed, laboratory settings can reproduce a working scenario in a controlled environment and they can be used for the preliminary testing of devices and new methods of evaluation, allowing for the use of technological instrumentations that provide objective measures to perform the biomedical assessment, such as kinematic parameters and EMG signals. However, real working scenarios are different and the worker can be affected by multiple factors that are not present in the simulated environment. Therefore, it is fundamental to directly assess the workers on the workplace during their usual working activity in order to monitor their physical state, so that the working experience can be improved, work-related musculoskeletal disorders can be prevented, and human compliant working environments can be arranged, improving ergonomics, workloads and sustainability. Therefore, we wanted to analyze in detail the differences between laboratory and workplace studies, and to investigate if the biomechanical assessments used in laboratories are being transferred to real working scenarios. Finally, this review also provides critical comments on the current state-of-the-art approaches and future trends and directions lying at the interdisciplinary intersection between biomechanics, ergonomics and human-centered approaches for the industrial field.

## 2. Materials and Methods

This research was designed to answer the main research question (RQ 0): “How have biomechanical assessments of the upper limb in an industrial context been implemented for assessing fatigue, strain and effort?”. RQ 0 is further split into the following research questions, addressed for both the laboratory and working settings:

RQ (1) Which topics were addressed and which findings were obtained?

RQ (2) Which kind of setting was used for the studies?

RQ (3) What type and how many participants were targeted and which anatomical target was assessed?

RQ (4) What type of motor tasks and protocol design were studied?

RQ (5) Which analysis techniques were employed?

We thus considered scientific articles that applied a biomechanical analysis, targeting industrial applications, and we provided an overview of the main topics addressed, and the setups considered for applications, exploring the data analysis techniques. The international guidelines established by PRISMA (Preferred Reporting Items for Systematic Reviews and Meta-Analyses) [21,22] were used.

### 2.1. Bibliographic Research Strategy

With the above-mentioned aims, the following procedure was employed for the literature screening. A collection of articles was obtained by screening Scopus and Web of Science (WOS), using a query based on the keywords: “shoulder”, “elbow”, “wrist”, “upper limb”, “upper extremity”, “arm”, “fatigue”, “effort”, “strain”, “workplace”, “industry”, “industrial”, “assessment”, “index”, “evaluation”, “biomechanics”, “measure”, “measurement” and all possible variants.

The formal logical query was (shoulder OR elbow OR wrist OR upper limb OR upper-limb OR upper extremity OR arm) AND (fatigue OR strain OR effort) AND (worker OR workplace OR industry OR industrial) AND (assessment OR index OR evaluation OR biomechanics OR measure OR measurement).

### 2.2. Eligibility Criteria and Study Selection

In the eligibility phase, we selected the articles relevant to the aim of this review. Screened articles had to satisfy all the following criteria:

(A) To include the terms selected in the above reported query in the abstract and/or title and/or in the keywords.
(B) To involve applications with biomechanical evaluations (in a broad sense, as this includes biomechanical models, EMG, and others).
(C) To target laboratory or workplace scenarios, with a clear aim at industrial applications.
(D) To be indexed in at least one of the screened databases.
(E) To be a full journal article.
(F) To be available in English.

The eligibility criterium was “criterium A AND criterium B AND criterium C AND criterium D AND criterium E AND criterium F”.

Lastly, some articles were also available before the year 2000. However, most of them regarded the use of obsolete technologies and methods, thus we decided not to include papers published before the year 2000. The screening was updated to the 31st of December 2022.

The selection process was performed with the papers screened one by one for inclusion by two different groups (made of subgroups of the authors of the review) independently. Each paper was screened by two different reviewers who blindly classified it as eligible or non-eligible. This allowed for a reduction in the risk of bias in the selection process. Any disagreement in the classification was settled by discussion between the two groups and a consensus was reached in all cases.

### 2.3. Data Extraction and Synthesis

Our review of the literature was organized to summarize the state-of-the-art information in the field by detailing the following categories:

#### 2.3.1. Main Topics and Findings

This section aimed to summarize the main topics addressed in the works and in the obtained results, answering the research question: “Which main topics were addressed and which findings were obtained?”. Since the studies were based on specific design and a wide variety of aims, we summarized the results dividing all the studies into categories.

*Basic research on biomechanical assessments in physiological conditions of fatigue:* includes studies in which physiological effects of fatigue were investigated in order to find fatigue indicators during working activities.

*Influence of task conditions on biomechanics:* includes studies that investigate how the presence of external loads, and the type and speed of movements can affect the biomechanics.

*Musculoskeletal diseases risk assessment:* includes studies that performed assessments in a variety of workplaces and jobs, in order to investigate the risk of developing musculoskeletal diseases (MSD).

*Effects of ergonomic interventions:* includes studies that proposed and evaluated interventions in order to mitigate and prevent pain and musculoskeletal injuries.

*Prevention and beneficial effects of exoskeletons/supporting devices:* includes studies that examined the effects of the use of exoskeletons or other supporting devices on biomechanics.

*Design and validation of assessment methods:* includes studies that proposed and validated alternative methods and more objective measures in comparison to the traditional scales and questionnaires.

*Protocols:* includes studies that proposed some protocols to be implemented in the following years.

#### 2.3.2. Setting

First of all, the studies were subdivided based on the setting, classified into “workplace”, “laboratory”, “simulated” and “protocol”. Then, all further analyses were performed dividing the selected papers in the following two categories: laboratory (that included also simulated and protocol studies that were considered as pilot work not performed on the field) and workplace.

#### 2.3.3. Type of Participants, Number of Participants, Anatomical Target

This section characterized the participants that were enrolled in the studies, answering the research question, “What type and how many participants were targeted and which anatomical target was assessed?”, and it is subdivided into:

*Type of participants:* this section answered the question: “Which type of participants were enrolled?”. The type of participants enrolled in the studies were divided into “volunteers”, “workers” and “simulated subjects” (by simulated subjects, we mean those made with biomechanical simulations and modelling).

*Number of participants:* this section specified the number of participants enrolled in each of the studies, answering the question “How many participants were enrolled?”.

*Anatomical target:* this section answered: “Which upper limb segments were considered in the paper?”, specifying the targeted upper limb segments for functional assessment in “proximal joints” (shoulder and elbow), “distal joints” (wrist and hand) or “both”, in order to better specify which upper limb joints were used in biomechanical evaluations.

#### 2.3.4. Tasks Type, Task Design, Task Support

This section described the design of the studies in terms of the characteristic of the motor tasks that were biomechanically analyzed, answering the research question “What type of motor tasks and protocol design were studied?”. This section was further divided into:

*Task type:* this section answered, “Which tasks were performed and analyzed?” and described the type of movements that were performed. We classified them into five main categories:

Lifting: studies in which participants were asked to lift weights as a primary task;

Postural: where participants were asked to hold a specific posture or train/test postural capabilities;

Functional: participants were asked to perform functional tasks that allow them to complete very specific goals;

Free: where participants could move freely in the environment, performing numerous tasks, in general and without clear or stringent constrains;

Others: all task types not belonging to the previous groups.

*Task design:* answered the question, “What was the design of the task and protocol?” and specifies the design of the task/protocol to be performed. We divided this section into three main groups:

Repetitive: participants performed the same task repetitively;

Controlled: participants performed tasks in specific constrained conditions;

Free: participants were not performing tasks with specific constrained conditions.

*Task support:* answered, “What supports were used to execute the tasks?” and specified which tools and devices were used to support the execution of the task. Supports were classified in 5 groups:

Free: tasks were performed without the use of specific tools;

Tool/handle: tasks required specific tools to be performed;

Exoskeleton/support: participants performed the task wearing exoskeletons or supporting devices;

Robot/end effector: tasks were performed in collaboration with a robotic end effector;

Others: tasks were performed with the use of devices not belonging to the previous categories.

#### 2.3.5. Type of Assessments

This section described which techniques and findings were used for the data analysis, answering the question: “Which analysis techniques have been employed?”. The assessments were classified into:

EMG: the assessment included muscle activity signals measured with EMG;

Kinematics: kinematic parameters, such as articular angles, velocities and accelerations, were used for the assessment;

Biomechanics/kinetics: biomechanical parameters, such as torques, power and energy, were used for the assessment;

Questionnaires/scales: the assessment was done mainly using questionnaires and scales, such as the OCRA Checklist [23], the Nordic Musculoskeletal Questionnaire (NMQ) [24], the Rapid Entire Body Assessment (REBA) [25] and others;

RULA: the Rapid Upper Limb Assessment [26] was used for the assessment; this is a scale method developed for use in ergonomics investigations of workplaces where work-related upper limb disorders are reported [27], and gives quantitative indexes based on directed measurements of articular angles.

Strain index: the Strain Index [28] was used for the assessment. It is a job analysis tool that uses both qualitative and quantitative methods to identify jobs that do and do not expose workers to an increased risk of developing a distal upper extremity (DUE) disorder [27].

Other measurements: other measures not included in the other categories, such as heart rate, Near Infrared Spectroscopy (NIRS) and electroencephalography (EEG).

## 3. Results

### 3.1. Study Selection

**Figure 1:**
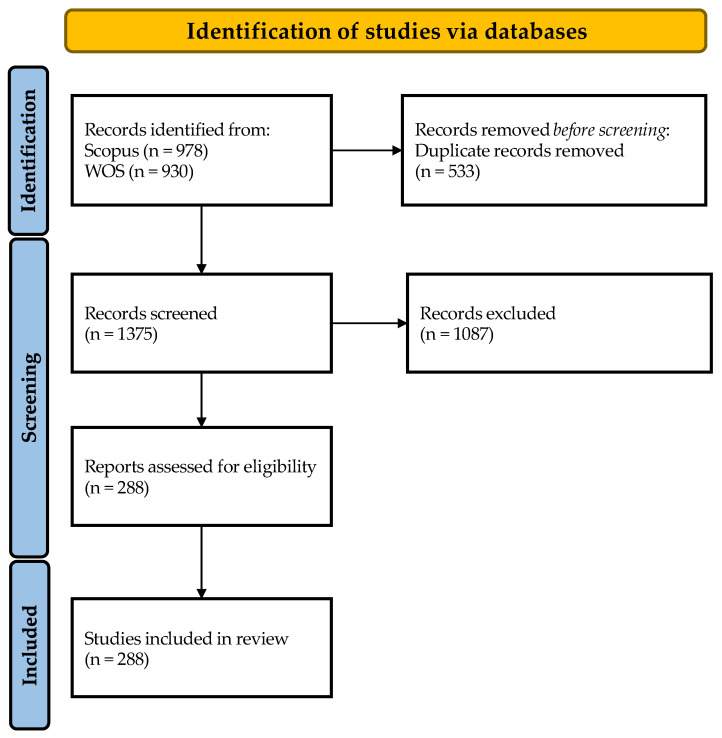
The PRISMA flow chart for the proposed literature review.

As a result of the screening, 978 papers were found on Scopus and 930 on Web of Science. The total number of articles was 1908 and, after duplicates removal, the number of screened articles was 1375. Papers that were not in English and conference papers were not considered. Due to non-adherence to the eligibility criteria, 79% of the papers were excluded (n = 1087). Reasons for the exclusion were: the not-targeting of industrial or workplace scenarios, but clinical or merely laboratory applications with not-foreseen further applications in industrial scenarios; the absence of any quantitative method for assessment, or data non-presented; the lack of crucial information, lack of data or evident incompleteness in the data or methodology presentation; non-full-text studies. After the screening phase, the number of papers identified as eligible, meeting all the selection criteria and included in the review, was 288. In the next sections, the results of our research are presented. The PRISMA flow chart summarizing all the steps for screening and inclusion is presented in Figure 1.

### 3.2. Assessing FSE: Main Findings

In this section, we briefly report the main findings of the studies screened (see Table 1) in the assessment of fatigue, strain or effort, or a combination of those. Due to their variability in aims and purposes, we reported the main topics of investigation aggregated according to categories that divided studies by topic, summarizing the main findings. The categories were ordered to highlight the specific features of each research aim, from basic research to practical applications, including protocols, application of ergonomic assessments and use of supporting devices.

#### 3.2.1. Basic Research on Biomechanical Assessments in Physiological Conditions of Fatigue

The physiological effects of fatigue during repetitive movements, overhead tasks and posture maintenance have been investigated in order to find indicators that can be used to identify fatigue during working activities. Joint angles and torques, especially at shoulder level, significantly reduce with fatigue [29,30]. Moreover, fatigue influences joint coordination that has to compensate for kinematic changes to maintain the trajectory of the end effector [31]. Systematic changes were found also on the power spectrum of the angular velocity and the acceleration of the shoulder and trunk [32]. Muscle fatigue is often detected with EMG signals, since spectral features such as the mean power frequency, the median frequency and the maximum voluntary contraction decrease with fatigue [33,34], clearly allowing the use of EMG as a biomarker for fatigue. Finally, the EMG signal can be associated with the EEG alpha band for the identification of mental and physical fatigue [35,36]. EMG and EEG signal coupling may provide a complete characterization of both the mental and physical state of the worker [317]. Indeed, physical and mental fatigue can be correlated in specific tasks, since the complexity and precision of the task increase fatigue [37].

#### 3.2.2. Influence of Task Conditions on Biomechanics

During working activities, workers usually interact with objects and tools, and perform movement in various directions. The presence of external loads and the direction of movements can affect the biomechanics and, consequently, induce fatigue. Moreover, in industrial assembly lines or interacting with devices, the work pace and movement velocity may be constrained and uncomfortable for the worker. Therefore, some studies investigated how biomechanics is affected by the task conditions, such as the presence of external loads, velocity and direction of movement, in order to provide ergonomic recommendations for new workstations. In general, additional, external loads increase the muscular workload and pain, and decrease the endurance time [81,101], suggesting that light tools should be preferred. The direction and height of the movement have significant effects on the muscular strain and body posture, increasing the overall discomfort, pain and fatigue [82]. Therefore, workstations should be designed in order to improve working postures, maintaining a posture that is as neutral as possible [96]. Finally, the work pace may affect the development of FSE. In fact, fast movements without rest decrease the oxygen saturation and muscle activity [90] and, therefore, a slow pace with rest should be preferred.

#### 3.2.3. Musculoskeletal Diseases Risk Assessment

Assessments during working activities were performed in a variety of workplaces and jobs, in order to investigate the risk of developing musculoskeletal diseases (MSD). In fact, physical risk factors at work and musculoskeletal disorders are associated with the increase of MSD [141]. The characterization of physical risk exposures of workers is needed in order to design tools and interventions capable of mitigating and, especially, preventing the development of MSD. Usually, scales and questionnaires such as REBA, RULA, SI and the OCRA checklist are used for risk evaluations since they can be administrated quickly on a large cohort of participants and they do not need specific architectures and equipment. However, some studies employed instrumental techniques, such as EMG, for the evaluation of the muscle activity during work and its correlation with MSD development [185]. Almost all the working activities analyzed resulted in having a high risk of MSD development, from industry and manual workers to office works, since these activities lead to high effort and strain. Moreover, repetitive activities [142] and high workloads [187] increase the risks of MSD. Therefore, ergonomic interventions are needed in order to correct working postures and to reduce pain and the risk of MSD [149].

#### 3.2.4. Effects of Ergonomic Interventions

Since most of the working activities are classified as being at high risk for MSD development, ergonomic interventions are needed in order to mitigate and prevent pain and musculoskeletal injuries. Some studies investigate if and how ergonomic tools and workstations can improve working postures and decrease the risk of MSD with respect to commercial and traditional ones, which are not tailored for specific needs. In general, ergonomic tools and workstations improved postures and comfort and reduced the strain, effort and, consequently, the risk of MSD occurrence and musculoskeletal complaints [243,251]. Better working conditions also resulted in increased productivity and quality [253]. However, workers may need time to familiarize with the new tools [249]. Moreover, ergonomic educational training making the worker aware of the risks and physical exercise programs showed improved working postures, reducing muscle activity and musculoskeletal complaints [214,239]. These interventions improve both physical and mental health [250] and are thus beneficial for workers [318].

#### 3.2.5. Prevention and Beneficial Effects of Exoskeletons/Supporting Devices

Repetitive tasks and posture maintenance (especially in overhead tasks) are one of the main causes of neck and back pain, and of joint load. Several exoskeletons and supportive devices, generally not actuated, have been developed to assist movements and postures during working activities. Exoskeletons reduce the muscles’ effort and joint load in the shoulder, arm and lower back [273,278], and this effect was proven in laboratory scenarios [258,272] and in preliminary campaigns conducted on field workers [263,265]. Thus, supporting devices reduce the EMG activity and fatigue, limiting the effects on joint torques and kinematics [270]. Moreover, the onset of muscular fatigue is delayed [280] and the oxygen consumption and heart rate are reduced [256]. Finally, the perceived effort and physical pain is reduced, improving the overall comfort of the worker [266,276].

#### 3.2.6. Design and Validation of Assessment Methods

A variety of assessment methods can be used for the evaluation of fatigue and strain during work. The most employed methods are scales, but other alternative methods and more objective measures are proposed and validated with comparisons to the traditional scales and questionnaires. The reliability of scales and the accordance between different methods were assessed, finding that the Strain Index is more specific for the distal upper limb evaluation, while the OCRA checklist allows for the assessment of the whole upper limb [308]. SI was also found to be more effective than RULA and REBA in non-fixed tasks [309]. Furthermore, the usability and reliability of technologies that allow for quantitative assessments were tested. The use of inertial measurement units (IMU) or Kinect cameras for fatigue assessments allow for the detection of kinematic changes in long-duration manual tasks [286]. These methods are easy-to-use in real time and could assist ergonomists in risk evaluations on site [306]. Moreover, the EMG signal can provide measures for detecting muscular fatigue and they can be correlated to kinematic and kinetic parameters to evaluate the global fatigue [293].

#### 3.2.7. Protocols

Some studies proposed protocols to be implemented in following studies. Three studies proposed new ergonomic intervention programs whose validity and efficacy will be tested with questionnaires and scales. Mathiassen et al. [313], instead, described an on-site biomechanical assessment, based on questionnaires and measurements of postures, movements and heart rate.

#### 3.2.8. Fatigue, Strain and Effort of the Upper Limb in Industrial Applications: Main Findings

**Figure 2:**
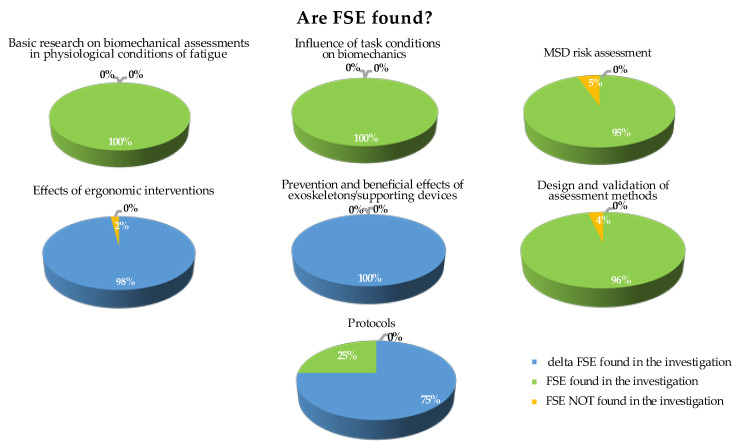
Main findings on FSE. For each category, papers were classified based on the main findings related to FSE. Papers showing differential effects of ergonomic interventions or supporting devices on FSE were classified in blue; papers that found FSE were classified in green; papers that measured but did not find FSE were classified in yellow.

We divided the papers into three groups depending on the main design and findings related to the assessment of fatigue, strain and effort. It is documented that FSE are a burden for the industrial field and for workers, as it was reported that three out of five workers in the European Union had MSD complaints due to their working activity [319]. On the basis of such epidemiologic data, some studies assumed that the effects of FSE are in most of the cases present and they are working to reduce such effects [258,273]. FSE are not directly measured, but rather technologies, protocols, devices, exoskeletons, methods, ergonomic platforms or interventions aim at reducing or preventing the effects of FSE. Therefore, often the assessments are based on differential measures and the main focus is on the reduction in the effects of FSE. Some studies, instead, directly measure when FSE are found. Most of the studies reported that fatigue is found in industrial tasks in working places [150,167] but also in simulated environments [29,32]. In a limited number of studies, the effects of FSE were not observed or they were under the limit for high risk of MSD development [117,136]. All the effects are summarized in Figure 2 of each of the categories of the study.

FSE are present in almost all the working activities, and represent a burden in the working scenario. Therefore, the assessment of FSE is important for preserving and improving workers’ health.

### 3.3. Setting

**Figure 3:**
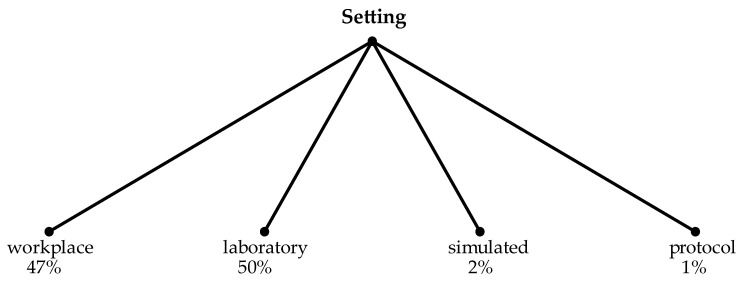
Distribution of settings in the screened articles.

The setting categories used in this review paper were: workplace, laboratory, simulated and protocol studies. A total of 50% of the works were performed in a laboratory environment; 47% of the works were performed directly in the workplace (or considering data relative to the workplace). Few studies were suggested/approved protocols (not yet implemented) or simulated studies. A visual representation of the settings is reported in Figure 3. The selected studies split almost equally into two groups: those made in laboratory environments, and those performed in working places. All the following assessments are based on the separation and comparison of these two groups clearly identified in the setting section. Simulated and protocol studies were considered as laboratory studies, since they do not involve workers and/or volunteers directly in the workplace.

**Figure 4:**
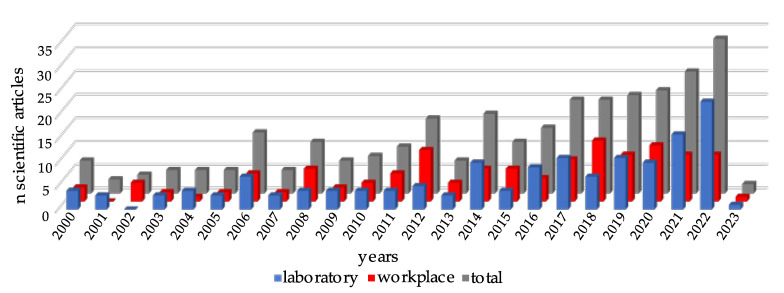
Temporal distribution (number of scientific articles published per year) of the screened scientific articles from the year 2000 to December 2022, for laboratory settings (blue), working places (red) and overall (grey). Some scientific articles that were already accepted and will be published in 2023 are available.

As shown in Figure 4, studies based on human-centered approaches for biomechanics of the upper limb in the industrial field are not new, even if the trend shows an increase in the papers published in the field in the last years (the screened papers are updated until the 31st of December 2022, with few papers already available and scheduled for publication for the year 2023). Interestingly, more recently, there has been an increase in the works based on laboratory settings, while the number of on-site works has stabilized. This trend could suggest that the interest is more focused on laboratory research activity than the translation of the assessments in the workplace. However, this finding should be commented in light of the fact that in the last three years, the restrictions due to COVID-19 may have impacted the on-field research.

### 3.4. Type of Participants, Number of Participants, Anatomical Target

#### 3.4.1. Type of Participants

**Figure 5:**
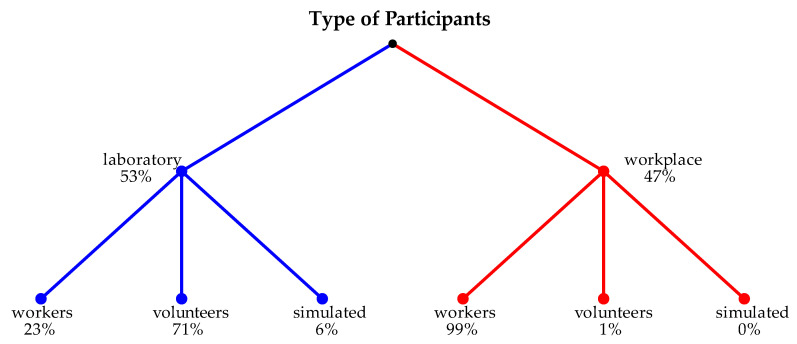
Distribution of selected papers based on the cohort of participants enrolled in the laboratory setting (**on the left**) and in the workplace setting (**on the right**). Participants were classified as workers, volunteers and simulated.

In the papers analyzed in this review, the cohorts of participants involved during the experimental sessions could be divided into 3 macro-categories: (i) volunteers, (ii) on-field workers and (iii) simulated subjects. In particular, 71% of the laboratory studies enrolled volunteers that did not have working experience related to the topic of the study and only 23% enrolled workers. In a minor number of laboratory studies (6%), the data were simulated starting from real recordings with biomechanical models (as in Brambilla et al. [109]) or completely simulated. Conversely, 99% of the workplace papers had enrolled workers. The categories of participants are graphically summarized in Figure 5. These results validate the choice to separate the laboratory and working places as they enrolled participants from different cohorts.

#### 3.4.2. Number of Participants

**Figure 6:**
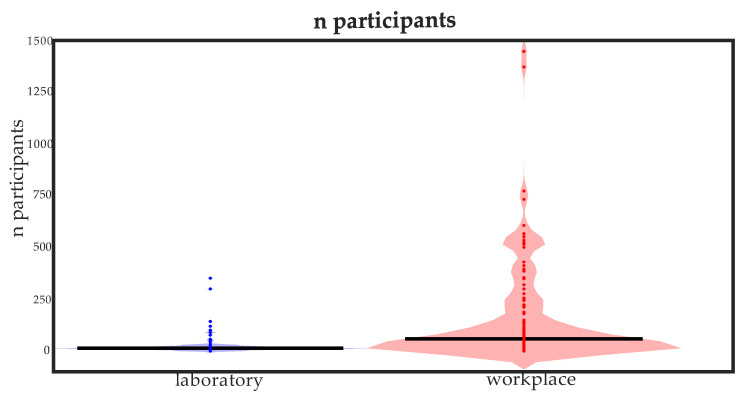
Distribution of the number of participants for a laboratory setting (**blue**) and for a workplace setting (**red**). The points indicate the value for each article and the black line is the median value. The number of participants (3141) of one workplace study is not shown in the figure for visualization purposes.

Considering the sample size, most of the laboratory studies involved less than 20 participants and only 10 papers involved more than 50 participants; the median number of participants was 14 and the 95th percentile was 68. In the workplace setting, most of the studies involved a high number of participants (>50) and 16 papers included more than 500 participants, with a maximum of 3141; the median number of participants was 62 and the 95th percentile was 560. Eight studies did not clearly declare the number of participants or presented protocols in which no participants were involved. A summary of the number of involved participants described in this section is reported in Figure 6.

**Figure 7:**
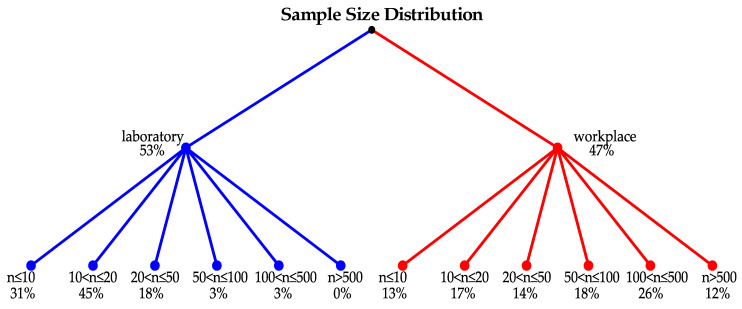
Sample size distribution in the laboratory setting (**on the left**) and in the workplace setting (**on the right**). The sample size was divided in six groups: n ≤ 10, 10 < n ≤ 20, 20 < n ≤ 50, 50 < n ≤ 100, 100 < n ≤ 500 and n > 500.

Figure 7 summarizes the sample size for both the laboratory and workplace papers. In a laboratory setting, most of papers included less than 20 participants (31% of the papers had less than 10 participants, 45% had a number of participants between 10 and 20); 18% of the studies included between 20 and 50 participants, while only 6% included more than 50 participants and none considered more than 500. The workplace design, instead, included various numbers of participants, uniformly distributed. The participants were less than 10 in 13% of the papers, between 10 and 20 in 17% of the cases and between 20 and 50 in 14% of the papers. About 56% of the studies included more than 50 participants, in particular: 18% involved between 50 and 100 participants, 26% between 100 and 500 participants, and 12% more than 500 participants.

#### 3.4.3. Anatomical Target

**Figure 8:**
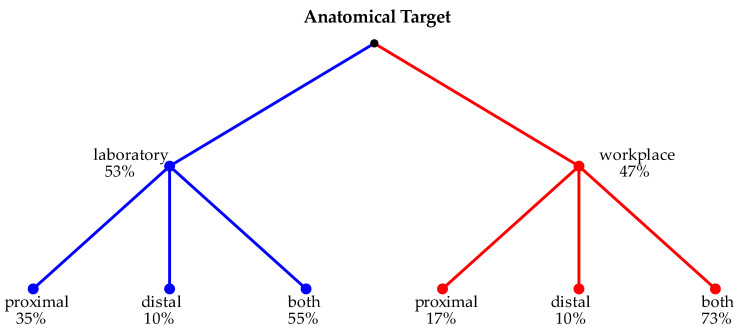
Anatomical targets considered in the laboratory setting (**on the left**) and in the workplace setting (**on the right**).

In Figure 8, a detailed representation of the anatomical targets for the considered studies were reported. In the laboratory studies, 35% reported an analysis in proximal joints, 10% reported an analysis mainly on distal joints, while most of the studies (55%) reported an analysis on both the anatomical targets. In the workplace studies, 17% reported a proximal analysis, 10% reported a distal analysis, and the majority (73%) reported an analysis on both the proximal and distal targets. There was a tendency toward extending the analyses to the whole upper limb in the workplace studies in order to perform a comprehensive assessment of the workers, while the laboratory studies showed more targeted investigations.

### 3.5. Task Type, Task Design, Task Support

#### 3.5.1. Task Type

**Figure 9:**
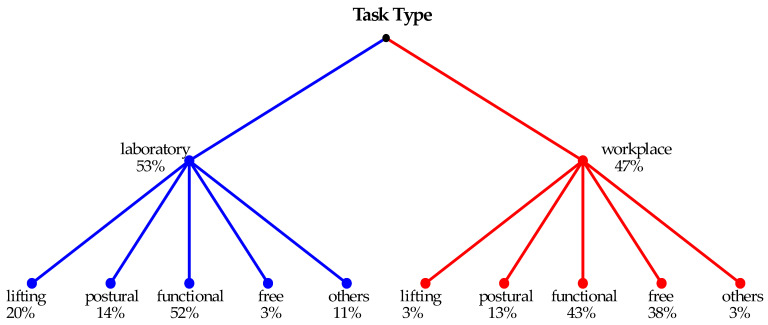
Percentage of the task types for the laboratory setting (**on the left**) and for the workplace setting (**on the right**).

Task type is reported in Figure 9. In the laboratory papers, most of the studies analyzed functional tasks (52%), followed by lifting tasks (20%) and postural tasks (14%); only 3% of the laboratory studies reported free movements. On the contrary, 38% of the workplace studies reported free movements and 43% functional movements. Only 3% of these papers regarded lifting tasks and 13% postural ones. In laboratory scenarios, the tasks generally reproduce specific movements of the working activities and, therefore, they could be precisely classified into categories. In workplace settings, instead, the tasks are functional movements when the participant performs only a specific task, while in the other cases, the workers perform multiple activities that are a combination of different functional subtasks.

#### 3.5.2. Task Design

**Figure 10:**
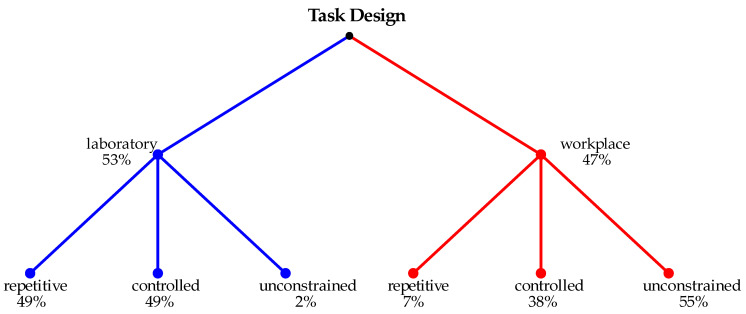
Percentage of the task design for the laboratory setting (**on the left**) and for the workplace setting (**on the right**).

Partially correlated with the task type, the task design showed that the laboratory studies were equally divided into repetitive (49%) and controlled (49%) movements that are by nature subject to experimental limitations, and only 2% of them considered unconstrained movements that represent more realistic working conditions in most of the cases. In the workplace studies, most of the studies (55%) considered unconstrained movements, 38% of the studies were based on a controlled design, and only 7% were conducted in repetitive conditions. The task design is reported in Figure 10.

#### 3.5.3. Task Support

**Figure 11:**
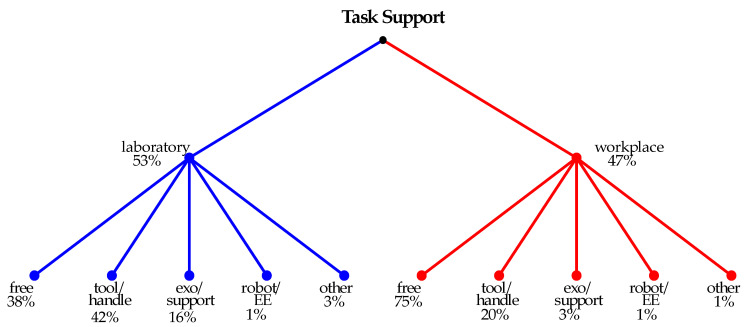
Percentage of the task supports employed for the laboratory setting (**on the left**) and for the workplace setting (**on the right**).

Supports are intended in a broad sense and they include devices and robots, but also tools and handles. Various kinds of task support were employed in the screened studies. Since most of the laboratory studies regarded the interaction with the environment and simulation of controlled tasks, 42% of them required tools and handles, including screwdrivers, hand supports, and others; 38% were based on free movements, while other supports (3%), end effector (EE) robots (1%) and exoskeletons (16%) were found in the other cases. In the workplace studies, participants usually performed their work during the entire workday, therefore the majority of them (75%) reported free movements, 20% used tools, while only 3% employed exoskeletons. The task support is reported in Figure 11. In the laboratory setting, there is a higher employment of tools and exoskeletons since new technologies, as new specific tools or supporting devices, are tested in a controlled environment, while in workplace scenarios, movements are generally free (as described in the task type), representing the whole working activity with multiple and various tasks.

### 3.6. Measurements and Data Analysis

**Figure 12:**
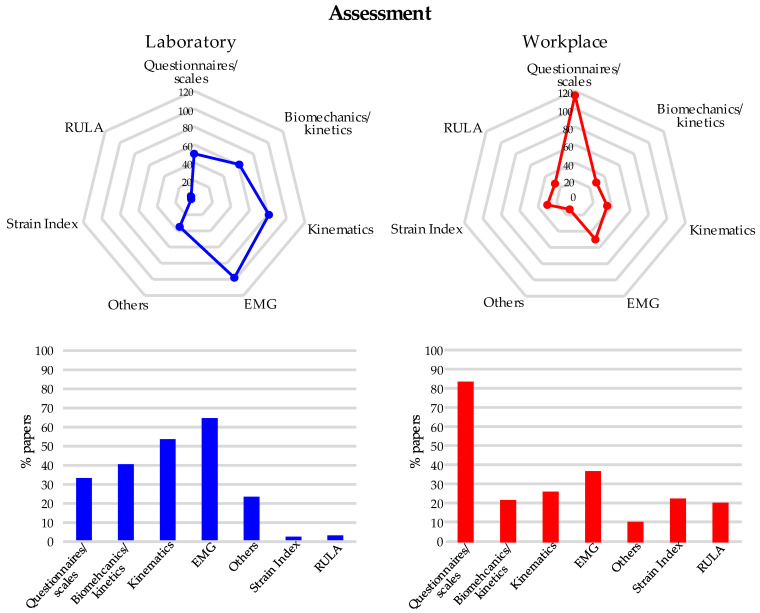
Assessment methods used in the considered studies. Some studies used more than one assessment (overall percentage could exceed 100%). In the upper panel, the number of studies that employed the considered assessment methods is reported for both laboratory ((**left panel**), in blue) and workplace settings ((**right panel**), in red). In the lower panel, the percentages of papers employing the assessment methods are shown in blue for laboratory and in red for workplace scenarios.

Several approaches were employed in the screened studies as shown in Figure 12. Some instrumental approaches were based on the EMG and kinematics, but also model-based approaches often included biomechanics and kinetics, with human models or recorded forces. Other approaches were based on scales and questionnaires. Some papers merged two or more of these approaches, even though usually sensors-based measures are used together as well as scales/questionnaires methods. Interestingly, the type of assessment differs consistently between the laboratory and workplace settings. In laboratories, EMG and kinematics are the most used methods to assess the biomechanics, effort, fatigue and strain, employed in more than 50% of the studies; on the contrary, in workplace settings, questionnaires and scales are by far the most employed ones (more than 80% of the studies).

## 4. Discussion

### 4.1. Summary of the Main Results

In this systematic review, we screened a large number of studies that performed biomechanical assessments to identify FSE in a working scenario. First, we found that a wide variety of topics were addressed by the screened studies: from the identification of physiological markers of fatigue, to the influence of task conditions on the biomechanics; from the MSD risk assessment during working activities to the design and definition of ergonomic interventions and to the testing of the effects of supporting devices, such as exoskeletons. In most of the studies, the working activities had high risk of MSD development and ergonomic interventions were needed. However, few works proposed practical solutions to solve such issues and concentrate more on assessment, while recent literature is leveraging on the validation of novel technologies. Moreover, several differences between studies taking place in laboratories and in workplaces were found. In a laboratory setting, healthy volunteers were principally included, performing movements in a controlled environment. In a workplace setting, instead, workers performing their usual working activities were analyzed, allowing for a more realistic assessment. However, this scenario limited the application of technologies that could provide the quantitative assessment of fatigue, and, therefore, scales and questionnaires were the most employed methods. In the laboratories, instead, EMG and kinematic measures were used for the assessment.

### 4.2. Rationale for a Top-Down Large Scope Screening on Fatigue, Strain and Effort

In this systematic review, we analyzed papers in which biomechanical assessments were used or analyzed for evaluating or assessing motor performance, fatigue, effort and strain in applications aimed at industrial scenarios, separating laboratory studies from those performed in the workplace. The integration of biomechanical assessments and physiological signals can be useful for the evaluation of fatigue, effort and strain in industrial scenarios, allowing for the investigation of the motor system, ergonomics and of mental health in their complexity, configuring a multi-disciplinary field of research at the intersection between several fields such as industry, biomechanics, ergonomic assessment, and medicine [320]. In our sample of articles, a wide variety of scenarios and assessments were found. The range of applications was very wide and non-homogeneous; while on one hand this variability complicated our analysis, on the other hand it allowed to perform a wide summary that allows for the generalization of trends in the field, and provide a comprehensive top-down view of the assessments available. Our large screening provides a comprehensive view of the actual context in which biomechanical assessments are performed in working applications and which are the following advancements that are needed for monitoring workers’ states during working activities. We summarized the results achieved so far, with an attempt to coordinate the available achievements and findings into homogeneous groups.

### 4.3. A Transition to a Human-Centered Perspective

From the distribution of selected papers over the years, 288 related studies emerged in our screening and they are in a growing trend, suggesting that the topic will expand further in the next years. Indeed, most studies were published in the last decade with a remarkable increase in number in the last 10 years. Increasing the mental health and the wellbeing of workers is becoming more and more of an emerging topic to improve the industrial field in a human-centered perspective [11]. The recent human-centered developments in the industrial field lead to a high level of automatization in order to increase the productivity and efficiency. In this way, human operators may face an increased complexity in their daily tasks with a higher physical and mental demand [321]. Therefore, the physical safety of workers is of primary importance [322] and the wellbeing of the industrial workers and the prevention of diseases with biomechanical assessments are a fundamental step to improve working conditions and reduce work-related musculoskeletal disorders [5]. This recent crucial step leads towards the investment of resources and research results mutating techniques, sensors and findings from the bioengineering field, in order to apply them to the industry to enhance the industrial environments in several ways.

These aspects can explain why the use of biomechanical analyses, or biomechanical-related measurements are rapidly becoming an emerging topic even outside of the medical field. This finding may also indicate that the human factor requires strong theoretical and technological improvements for adapting methods and technologies from bioengineering and mechanical engineering for use in the industrial field, thus fostering laboratory investigations. This human-centered revolution includes the introduction of assistive devices and exoskeletons that are topical for many applications in the field [17]; at the moment, it is limited mainly to devices but is growing also in the direction of employment of techniques for motor control [323,324], bio-signal analysis with advanced techniques from bioengineering [325], bio-inspired control [326], fatigue detection and others.

### 4.4. Main Findings on FSE of the Upper Limb

Given the high variability in the aims, the studies were divided based on the main topic of their findings. One category included all the papers that investigated the physiological effects of FSE during the simulated and constrained movements. They found that the presence of FSE can be detected by changes in the kinematics [32], dynamics [30] and also in the EMG signal [33]. Papers that analyzed the influence of the movement conditions, such as the presence of external loads [101], velocity and direction of movement [82], on biomechanics providing ergonomic recommendations for preventing fatigue, strain and effort were grouped in another category. As in the previous category, these articles directly measured and detected FSE and provided recommendations that could be used for improving the workstation design. The largest category consisted of papers that assessed the risk of MSD development, identifying most of the working activities as having a high risk, since the FSE exceeded the recommended threshold limits. These assessments were performed mainly with scales and questionnaires [193,202], but in some cases also kinematics and EMG signals were used [135,167]. Very few studies did not find FSE in the working tasks [117,136]. Another group of studies identified ergonomic interventions, such as new ergonomic workstations [243], tools [229] or training programs [239] that could be implemented in order to improve the working postures and reduce FSE. Other studies proposed exoskeletons or supporting devices in order to reduce the FSE, especially in prolonged posture maintenance and overhead working tasks [259,263] that require high loads on the neck, back and shoulder [261]. Another category included papers that design and validate alternative methods for assessing FSE during working activities, such as marker-less systems [286,306] or EMG measures [293]. Finally, few papers described new protocols for assessing FSE or for implementing intervention programs.

From this analysis, two research lines emerged: one consisted of the investigation of FSE from a physiological point of view and in their identification during working activities; the other one proposed and tested technologies and instruments aimed at reducing and preventing FSE. It is important to foster the research on mechanisms and factors influencing the development of FSE. However, since the majority of the working activity resulted in tasks with the presence of fatigue and at high risk of MSD development, the development of new ergonomic solutions to be actuated in order to reduce and prevent FSE should become of primary interest. Moreover, in the human-centered perspective of Industry 5.0, the safety and physical state of the worker has become a primary driver for future developments in the field [5]. In this scenario, new methods and technologies need to be implemented with practical applications [8]. Moreover, raising awareness and promoting education among workers on the risks of MSDs is an important element for improving their physical health [327]. Studies demonstrated that educational training reduced the biomechanical exposure and the musculoskeletal symptoms in the neck and upper limbs [328,329]. Finally, early ergonomic interventions may prevent the development of MSDs and pain in workers [330].

### 4.5. Laboratory vs. Working Setting

We found that studies in laboratories and workplaces are about of the same number, with an unexpected recent trend promoting laboratory investigations. This result could indicate a higher interest in the research activity or in testing new devices and methods instead of assessments in the workplaces, and a consequent push toward novel technological innovation. Another factor that may explain the increase in laboratory studies in recent years is related to the restrictions due to COVID-19 pandemics, that limited the working activities and also on-site assessments. Moreover, we found that many studies taking place in laboratories were observational or pilot studies—mainly focused on preliminary works in which novel experimental setups or concept designs were tested on a limited number of participants. Only some papers presented structured, comprehensive investigations that evaluated the fatigue, effort and strain in detail in large cohorts of people with the aim of extracting results that could be generalized to a large sample of people. On the contrary, in workplace settings many more studies assessed large cohorts of participants, but they employed fairly rapid and subject-dependent assessments (such as questionnaires or scales). One study could screen the impressive number of 3141 participants [181]. In particular, the studies taking place in the laboratory included principally only healthy participants performing constrained movements in order to examine the physiological effects of fatigue or to test new devices. This partially limits the range of application of the results, since the assessments are not fully adherent to real workers’ activities; on the contrary, they show a major technological push to introduce new techniques and technologies to comply to the requirements of the field. In workplace settings, only workers were recruited and they performed their usual working activity. However, the real working scenario reduced the availability of a detailed biomechanical assessment since some technologies cannot be easily used in uncontrolled environments. In fact, ~80% of the studies in the workplace setting employed scales and questionnaires for the assessment. However, these kinds of assessment are not completely objective since they may depend on the subjective sensations of the worker and also on the rater that administers the scale. Detailed instrumental analyses were instead performed mainly in the studies with participants. The high number of the pilot and observational studies indicated that the use of complex approaches and techniques was found mainly in the studies that aimed at exploring novel research purposes rather than deepening topics in detail with generalization objectives. This is understandable considering the feasibility of some approaches that require complex setups for data gathering, which are not always compatible with working activities, high costs, invasive setups or time-consuming procedures. Consequently, it arose from the literature that there is a relevant trade-off between the papers that deal with a large number of workers, and especially working scenarios, and the assessments that were performed. It followed that more time-consuming techniques were mostly employed in preliminary studies for evaluating how some protocols or assessments are accepted by workers or are useful to determine their level of FSE.

### 4.6. Translating Biomechanical Assessments from Laboratory to the Workplace

From the screening of the papers, the studies taking place in laboratories were principally observational or were pilot studies in which new tools and supporting devices were tested or some biomarkers for FSE were identified [29,32,33]. Future directions should foresee more structured and comprehensive studies involving large cohorts of participants, developed starting from pilot studies already available so that more reliable conclusions can be drawn. The laboratory studies enrolling healthy participants should be used as a benchmark for assessing the physical state of the worker and for identifying the pathological changes occurring in disorders that may occur for workers [93]. Moreover, we foresee for future developments that the environment will be less and less controlled, focusing in more detail on the real interaction of workers with their workplaces. This should be mixed with detailed biomechanical assessments also including kinematic, EMG, kinetic and dynamic measurements that can better complement and specify with more detail—and continuously monitor—the findings of the scales/questionnaire’s assessments. Indeed, the laboratory research should be translated into the real working scenario, using objective measures for the assessments, instead of scales only [307].

Another relevant issue not always reported in the studies is how the findings and the assessments can relevantly enter into the working practice. In laboratory settings, the proposed tools and experimentations show some biomarkers for fatigue and strain; there should be suggested methods and devices for reducing the workload in simulated working tasks or in quasi-static postures [272]. However, the application to the real working scenario may show different results and, therefore, it is necessary to apply the new technologies in the workplace to assess the real efficacy [331]. In the workplace setting, instead, lots of studies showed that many activities performed during work required high strain and effort and workers might develop musculoskeletal disorders related to their job. However, very few works suggest how these situations can be changed and how the effects of ergonomic interventions are generally verified with scales. Future directions should design solutions for improving the worker’s wellbeing and validate their efficacy not only in a controlled laboratory environment but also in the real workplace.

## 5. Conclusions

In this systematic review, we provided a wide screening of studies that performed biomechanical assessments in order to identify fatigue, strain and effort during work. First of all, we found that a wide variety of topics are addressed when performing biomechanical assessments in industrial scenarios. The studies suggested that most of the working activities are at high risk of MSD development and that ergonomic interventions are needed. However, few works proposed changes that can be done with ergonomic workstations and the use of supporting devices. Moreover, we found that in laboratory settings, the studies included principally healthy volunteers that performed movements in a controlled environment that replicated the workplace. In workplace settings, instead, workers were recruited and assessed during their usual working activity. This allows for a real assessment but limits the application of technologies that provide a quantitative assessment of fatigue. Therefore, in a human-centered perspective, the translation of new technological assessments into the real practice is needed to improve the comprehension and devise new ways to protect the physical and mental health of the worker.

## Figures and Tables

**Table 1:** Summary of the main findings, divided into categories related to the topic of the investigation.

Category	N Articles	Main Outcomes	References
Basic research on biomechanical assessments in physiological conditions of fatigue	48	Fatigue has effects on joint kinematics, torques and coordination Fatigue increases power spectrum of velocity and acceleration Muscle fatigue can be detected with EMG signal Duration, complexity and precision of the task increase muscle fatigue The EMG signal associated with the EEG alpha band can identify mental and physical fatigue	[29,30,31,32,33,34,35,36,37,38,39,40,41,42,43,44,45,46,47,48,49,50,51,52,53,54,55,56,57,58,59,60,61,62,63,64,65,66,67,68,69,70,71,72,73,74,75,76]
Influence of task conditions on biomechanics	39	Loads increase the muscular workload and fatigue and pain, and decrease the endurance time Direction and height of movement have significant effects on muscular strain and body posture Higher work pace decreases the oxygen saturation and increases muscle activity	[77,78,79,80,81,82,83,84,85,86,87,88,89,90,91,92,93,94,95,96,97,98,99,100,101,102,103,104,105,106,107,108,109,110,111,112,113,114,115]
MSD risk assessment	92	Association between physical risk factors at work and MSD REBA, RULA, Strain index, OCRA checklist can identify working posture at risk for development of MSD Repetitive movements and high workloads increase the risk of MSD Ergonomic interventions are needed to reduce the risk of developing MSD	[116,117,118,119,120,121,122,123,124,125,126,127,128,129,130,131,132,133,134,135,136,137,138,139,140,141,142,143,144,145,146,147,148,149,150,151,152,153,154,155,156,157,158,159,160,161,162,163,164,165,166,167,168,169,170,171,172,173,174,175,176,177,178,179,180,181,182,183,184,185,186,187,188,189,190,191,192,193,194,195,196,197,198,199,200,201,202,203,204,205,206,207]
Effects of ergonomic interventions	47	New ergonomic tools/workstations improve postures and comfort with respect to traditional or commercial ones and reduce the risk of MSD occurrence and musculoskeletal complaints Better working conditions result in a higher productivity Ergonomics interventions improve working postures, decreasing the occurrence of MSDs Physical exercise programs can reduce muscle activity and musculoskeletal complaints Ergonomic interventions can reduce physical and mental fatigue	[208,209,210,211,212,213,214,215,216,217,218,219,220,221,222,223,224,225,226,227,228,229,230,231,232,233,234,235,236,237,238,239,240,241,242,243,244,245,246,247,248,249,250,251,252,253,254]
Prevention and beneficial effects of exoskeletons/supporting devices	30	Exoskeletons reduce the muscles’ effort, joint load and global fatigue in overhead activities Onset of muscular fatigue is delayed Supporting device reduces the effects of fatigue on kinematics Exoskeleton reduces the oxygen consumption and heart rate	[255,256,257,258,259,260,261,262,263,264,265,266,267,268,269,270,271,272,273,274,275,276,277,278,279,280,281,282,283,284]
Design and validation of assessment methods	28	SI is specific for distal upper limb evaluation, while OCRA evaluates the whole upper limb IMU, Kinects and EMG can be used to measure physical demand in workplace assessments New scales and methods are validated comparing the results with traditional scales or assessments by experts	[285,286,287,288,289,290,291,292,293,294,295,296,297,298,299,300,301,302,303,304,305,306,307,308,309,310,311,312]
Protocols	4	New intervention programs are proposed to reduce MSD risk A biomechanical assessment based on questionnaires and kinematic measures is proposed	[313,314,315,316]

## Data Availability

No new data were created or analyzed in this study. Data sharing is not applicable to this article.
